# Supplementary material for: A discrete relativistic spacetime formalism for 1 + 1-QED with continuum limits
Source: Sci Rep. 2022 Feb 9;12:2198. doi: 10.1038/s41598-022-06241-4 (PMC8828844; doi:10.1038/s41598-022-06241-4)
Supplement: Supplementary file 1 — Supplementary Information. [file 41598_2022_6241_MOESM1_ESM.pdf]

Supplementary Material

1/-Proof of commutation rules and qubit gates as fermionic operators

We used a qubit to fermion transformation in order to simplify computations and be able to compare easily with the Schwinger Hamiltonian. In this appendix, we show that we indeed defined fermions by verifying the commutation rules and also express qubit matrices as fermionic operators.

$$\phi_p = ...(I)_{p-1} \otimes (E_{01})_p \otimes (\sigma_z)_{p+1}...$$
$$\phi_p^\dagger = ...(I)_{p-1} \otimes (E_{10})_p \otimes (\sigma_z)_{p+1}...$$

a) $\{\phi_p, \phi_{p'}^\dagger\} = \delta_{p,p'} I$
If $p = p'$

$$\phi_p \phi_p^\dagger + \phi_p^\dagger \phi_p = ...(I)_{p-1} \otimes (\underbrace{E_{01}E_{10}}_{E_{00}})_p \otimes (I)_{p+1}... + ...(I)_{p-1}(\underbrace{E_{10}E_{01}}_{E_{11}})_p \otimes (I)_{p+1}... = I,$$

if $p \neq p'$ (we choose $p' > p$)

$$\phi_p \phi_{p'}^\dagger + \phi_{p'}^\dagger \phi_p = ...(I)_{p-1} \otimes (E_{01})_p \otimes (\sigma_z)_{p+1}...(\underbrace{\sigma_z E_{10}}_{-E_{10}})_{p'} \otimes (I)_{p'+1}...$$
$$+ ...(I)_{p-1} \otimes (E_{01})_p \otimes (\sigma_z)_{p+1}...(\underbrace{E_{10}\sigma_z}_{E_{01}})_{p'} \otimes (I)_{p'+1}.... = 0,$$

Combining the results gives us the anticommutation rule for ϕ_p.
The same computation can be done for $\{\phi_p, \phi_{p'}\} = 0$ and $\{\phi_p^\dagger, \phi_{p'}^\dagger\} = 0$.

Let us now verify that the fermonic version of the qubit operators $\tilde{W}_p$ we proposed is correct by proving the following equality :

$$\tilde{W}_p = \begin{pmatrix} 1 & 0 & 0 & 0 \\ 0 & e^{-i\zeta}\sin\theta & \cos\theta V_{p+\frac{1}{2}} & 0 \\ 0 & -\cos\theta V_{p+\frac{1}{2}}^\dagger & e^{i\zeta}\sin\theta & 0 \\ 0 & 0 & 0 & 1 \end{pmatrix} = \phi_p\phi_p^\dagger\phi_{p+1}\phi_{p+1}^\dagger + e^{-i\zeta}\sin\theta\,\phi_p\phi_p^\dagger\phi_{p+1}^\dagger\phi_{p+1} + e^{i\zeta}\sin\theta\,\phi_p^\dagger\phi_p\phi_{p+1}\phi_{p+1}^\dagger$$
$$-\cos\theta V_{p+\frac{1}{2}}\phi_p\phi_{p+1}^\dagger - \cos\theta V_{p+\frac{1}{2}}^\dagger\phi_p^\dagger\phi_{p+1} + \phi_p^\dagger\phi_p\phi_{p+1}^\dagger\phi_{p+1}. \tag{1}$$

We verify each term of the sum by replacing the fermionic operators by their qubit definition :

$$\phi_p\phi_p^\dagger\phi_{p+1}\phi_{p+1}^\dagger = ...(I)_{p-1}(\underbrace{E_{01}E_{10}}_{E_{00}})_p(\sigma_z^2\underbrace{E_{01}E_{10}}_{E_{00}})_{p+1}\otimes(\sigma_z^4)_{p+2}... = ...(I)_{p-1}(E_{00})_p(E_{00})_{p+1}\otimes(I)_{p+2}...$$

$$e^{-i\zeta}\sin\theta\phi_p\phi_p^\dagger\phi_{p+1}^\dagger\phi_{p+1} = e^{-i\zeta}\sin\theta(\underbrace{E_{01}E_{10}}_{E_{00}})_p(\sigma_z^2\underbrace{E_{10}E_{01}}_{E_{11}})_{p+1} = e^{-i\zeta}\sin\theta(E_{00})_p\otimes(E_{11})_{p+1}$$

$$e^{i\zeta}\sin\theta\phi_p^\dagger\phi_p\phi_{p+1}\phi_{p+1}^\dagger = e^{i\zeta}\sin\theta(\underbrace{E_{10}E_{01}}_{E_{11}})_p(\sigma_z^2\underbrace{E_{01}E_{10}}_{E_{00}})_{p+1} = e^{i\zeta}\sin\theta(E_{11})_p(E_{00})_{p+1}$$

$$-\cos\theta\phi_p\phi_{p+1}^\dagger V_{p+\frac{1}{2}} = -\cos\theta(E_{01})_p(\underbrace{\sigma_z E_{10}}_{-E_{10}})_{p+1}V_{p+\frac{1}{2}} = \cos\theta(E_{01})_p\otimes(E_{10})_{p+1}V_{p+\frac{1}{2}}$$

$$-\cos\theta\phi_p^\dagger\phi_{p+1}V_{p+\frac{1}{2}}^\dagger = -\cos\theta(E_{10})_p(\underbrace{\sigma_z E_{01}}_{E_{01}})_{p+1}V_{p+\frac{1}{2}}^\dagger = -\cos\theta(E_{10})_p\otimes(E_{01})_{p+1}V_{p+\frac{1}{2}}^\dagger$$

$$\phi_p^\dagger\phi_p\phi_{p+1}^\dagger\phi_{p+1} = (\underbrace{E_{10}E_{01}}_{E_{11}})_p(\sigma_z^2\underbrace{E_{10}E_{01}}_{E_{11}})_{p+1} = (E_{11})_p(E_{11})_{p+1},$$

2/-Gauge invariance

We want to show the following equality :

$$P_\varphi G = GP_\varphi, \tag{2}$$

Let us begin with the computation of GP_φ. First we define a gauge field background. To do so, we have to give a value for each links on the lattice. Let's assume that each link has a value labeled by its position, therefore the state of the gauge field is : $\bigotimes_p |l_{p+\frac{1}{2}}\rangle$.

Now we put a particle at a site $2p$ (left-moving), the state of the whole system reads :

$$|...0_{2p-1}l_{2p-\frac{1}{2}}1_{2p}l_{2p+\frac{1}{2}}0_{2p+1}...\rangle\,. \tag{3}$$

We compute the phase θ produced by $P_{\varphi(x)}$:

$$\theta = ... + (l_{2p-\frac{3}{2}} - l_{2p-\frac{1}{2}})\varphi(2p-1) + (l_{2p-\frac{1}{2}} + 1 - l_{2p+\frac{1}{2}})\varphi(2p) + (l_{2p+\frac{1}{2}} - l_{2p+\frac{3}{2}})\varphi(2p+1) + ... \tag{4}$$

The state is now :

$$e^{i\theta}|...0_{2p-1}l_{2p-\frac{1}{2}}1_{2p}l_{2p+\frac{1}{2}}0_{2p+1}...\rangle\,. \tag{5}$$

Applying G (keeping only the states, not the amplitudes in front), we find :

$$\begin{aligned} &e^{i\theta}|l_{2p-\frac{1}{2}}1_{2p}l_{2p+\frac{1}{2}}\rangle \to W^*W \to e^{i\theta}\Big[|l_{2p-\frac{1}{2}}1_{2p}l_{2p+\frac{1}{2}}\rangle + |1_{2p-1}(l_{2p-\frac{1}{2}}+1)0_{2p}l_{2p+\frac{1}{2}}\rangle \\ &+ |l_{2p-\frac{1}{2}}0_{2p}(l_{2p+\frac{1}{2}}-1)1_{2p+1}\rangle + |1_{2p-2}(l_{2p-\frac{3}{2}}+1)0_{2p-1}(l_{2p-\frac{1}{2}}+1)0_{2p}l_{2p+\frac{1}{2}}\rangle\Big]. \end{aligned} \tag{6}$$

This gives us the state after GP_φ, now we do the same with the left-hand side of (2), that is $P_\varphi G$. We first apply G and get the state :

$$|l_{2p-\frac{1}{2}}1_{2p}l_{2p+\frac{1}{2}}\rangle + |1_{2p-1}(l_{2p-\frac{1}{2}}+1)0_{2p}l_{2p+\frac{1}{2}}\rangle + |l_{2p-\frac{1}{2}}0_{2p}(l_{2p+\frac{1}{2}}-1)1_{2p+1}\rangle + |1_{2p-2}(l_{2p-\frac{3}{2}}+1)0_{2p-1}(l_{2p-\frac{1}{2}}+1)0_{2p}l_{2p+\frac{1}{2}}\rangle\,. \tag{7}$$

We now apply the gauge transformation on each of the four states.e find θ for each. We put the first term as an example :

$$... + (l_{2p-\frac{3}{2}} - l_{2p-\frac{1}{2}})\varphi(2p-1) + (l_{2p-\frac{1}{2}} + 1 - l_{2p+\frac{1}{2}})\varphi(2p) + (l_{2p+\frac{1}{2}} - l_{2p+\frac{3}{2}})\varphi(2p+1) + ... = \theta, \tag{8}$$

Therefore, the four states have the same phase θ, we can factorize it and find the state to be :

$$e^{i\theta}\Big[|l_{2p-\frac{1}{2}}1_{2p}l_{2p+\frac{1}{2}}\rangle+|1_{2p-1}(l_{2p-\frac{1}{2}}+1)0_{2p}l_{2p+\frac{1}{2}}\rangle \\ +|l_{2p-\frac{1}{2}}0_{2p}(l_{2p+\frac{1}{2}}-1)1_{2p+1}\rangle+|1_{2p-2}(l_{2p-\frac{3}{2}}+1)0_{2p-1}(l_{2p-\frac{1}{2}}+1)0_{2p}l_{2p+\frac{1}{2}}\rangle\Big], \tag{9}$$

which is exactly the state found for GP_φ.
We can do the same for a particle beginning at $2p-1$ (right-moving) : $|l_{2p-\frac{3}{2}}1_{2p-1}l_{2p-\frac{1}{2}}\rangle$. The phase before applying G is

$$\theta' = ...+(l_{2p-\frac{5}{2}}+1-l_{2p-\frac{3}{2}})\varphi(2p-2)+(l_{2p-\frac{3}{2}}+1-l_{2p-\frac{1}{2}})\varphi(2p-1)+(l_{2p-\frac{1}{2}}-l_{2p+\frac{1}{2}})\varphi(2p)+... \tag{10}$$

If we apply G before P_φ we find the same phase.
In the end, we can conclude that

$$\boxed{P_\varphi G = GP_\varphi}, \tag{11}$$

the model is thus gauge-invariant under the $U(1)$ gauge group in a sense appropriate for Quantum Cellular Automaton.
